# Wind gradient exploitation during foraging flights by black skimmers (*Rynchops niger*)

**DOI:** 10.1242/jeb.246855

**Published:** 2024-08-22

**Authors:** Laura X. Mendez, Tyson L. Hedrick

**Affiliations:** Department of Biology, University of North Carolina at Chapel Hill, Chapel Hill, NC 27599, USA

**Keywords:** Kinematics, Flight angle, Energy saving, Wind effectiveness, Groundspeed, Airspeed

## Abstract

Birds commonly exploit environmental features such as columns of rising air and vertical windspeed gradients to lower the cost of flight. These environmental subsidies may be especially important for birds that forage via continuous flight, as seen in black skimmers. These birds forage through a unique behavior, called skimming, where they fly above the water surface with their mandible lowered into the water, catching fish on contact. Thus, their foraging flight incurs costs of moving through both air and water. Prior studies of black skimmer flight behavior have focused on reductions in flight cost due to ground effect, but ignored potential beneficial interactions with the surrounding air. We hypothesized a halfpipe skimming strategy for skimmers to reduce the foraging cost by taking advantage of the wind gradient, where the skimmers perform a wind gradient energy extraction maneuver at the end of a skimming bout through a foraging patch. Using video recordings, wind speed and wind direction measurements, we recorded 70 bird tracks over 4 days at two field sites on the North Carolina coast. We found that while ascending, the skimmers flew more upwind and then flew more downwind when descending, a pattern consistent with harvesting energy from the wind gradient. The strength of the wind gradient and flight behavior of the skimmers indicate that the halfpipe skimming strategy could reduce foraging cost by up to 2.5%.

## Teaser

**Summary:** Black skimmers can gather energy from the vertical wind gradient to help subsidize the flight cost of their unique feeding behavior.

## INTRODUCTION

Birds use different strategies to lower the cost of flight (Portugal et al., 2014; Usherwood, 2016; Weimerskirch et al., 2001). Among these strategies we find exploitation of different aspects of the environment to subsidize the cost of flight and reduce the need for use of muscle-powered flapping. Birds are known to gain energy by riding the rising columns of hot air in what is known as thermal soaring. This strategy saves energy during foraging flights, especially for larger species where flapping flight would present a costly endeavor (Bohrer et al., 2012; Mohamed et al., 2022; Shepard et al., 2011; Weimerskirch et al., 2003), but thermal soaring is also seen in smaller species capable of sustained flapping flight (Ruaux et al., 2023). Other species take advantage of the updraft resulting from orographic lift to clear high obstacles, or undertake long migrations at lower costs (Bishop et al., 2015; Bohrer et al., 2012; Mohamed et al., 2022) and in a smaller scale, but in a similar way, using the updraft from waves at sea (Stokes and Lucas, 2021). Birds even use small scale changes in air flow and vertically directed flows that occur around objects such as buildings as an energy source (Scacco et al., 2019; Shepard et al., 2016). These examples all rely on gathering energy from upwardly moving air, but birds also exploit other types of atmospheric energy.

**List of symbols and abbreviations**

*K*_a_: mass-specific kinetic energy in the air reference frame
*K*_g_: mass-specific kinetic energy in the ground reference frame
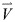
observed bird velocity in the ground reference frame
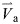
bird velocity in the air reference frame
*V*_a_: bird airspeed
*V*_g_: bird groundspeed
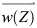
wind vector as a function of altitude
*Z*: distance over water surface (altitude), vertical axis
*Z′*: vertical speed
α: wind shear exponent
γ: bird flight trajectory angle in the horizontal plane
ε: wind effectiveness number
ψ_g_: flight angle with respect to the wind in the ground reference frame
ψ_a_: flight angle with respect to the wind in the air reference frame

Birds traveling and foraging over open waters take advantage of the vertical wind gradient over the water surface via dynamic soaring (Kempton et al., 2022; Mohamed et al., 2022; Sachs, 2005). During albatross gliding flight, and during flap-gliding flight in other Procellariiformes, dynamic soaring birds can experience a positive energy expense (Pennycuick, 1982). In dynamic soaring, birds extract energy from the vertical wind gradient by varying their flight direction relative to the wind direction such that they ascend while flying into the wind, increasing their kinetic energy with respect to the air reference frame, or descend while flying downwind, again increasing their kinetic energy in the air reference frame as they transition from faster to slower wind speeds (Denny, 2008; Mohamed et al., 2022; Sachs, 2005). On-bird dataloggers demonstrated that albatrosses can cover vast distances with minimal flapping flights via dynamic soaring, but conclusively demonstrating that dynamic soaring in flapping birds becomes challenging (Kempton et al., 2022).

Taking advantage of environment energy to subsidize flapping flight becomes increasingly important for foraging flights under conditions that could increase costs, such as movements between different fluids as at the air–water interface (Aigeldinger and Fish, 1995; Crandell et al., 2019; Gough et al., 2015; Green et al., 2009), even if the available subsidy cannot fully cover the cost of flight. One unusual foraging behavior that falls within these characteristics is displayed by the skimmers (genus *Rynchops*). The North American black skimmers (*Rynchops niger*) are coastal birds found along the East and West coasts of North and Central America, and the Caribbean (Vieira et al., 2018). These birds use a tactile form of feeding that allows them to exploit surface-dwelling fish found in calm and shallow waters, often at low tide (Vieira et al., 2018). They immerse and drag their modified mandible (elongated and laterally compressed resembling a sharp knife; Zusi, 1962), through the water as they fly close to the surface over a feeding patch, and when they make contact with prey, they rapidly close their bill and swallow the prey in flight (Withers and Timko, 1977; Zusi, 1962). This feeding behavior is referred to as skimming. Classic biomechanical and ecophysiological analysis of feeding by skimming flight (Blake, 1985; Withers and Timko, 1977; Zusi, 1962) suggests that its viability depends on another interaction with the environment: flight in ground effect, where the presence of a surface just below the wings of the animal reduces the induced drag generated by lift production, reducing the cost of flight (Blake, 1983; Hainsworth, 1988; Johansson et al., 2018; Rayner, 1991). Without this cost reduction, feeding by skimming might require more energy than it returns from capturing prey (Blake, 1985).

The foraging behavior of black skimmers has been studied previously in the context of foraging time and diet (Black and Harris, 1983; Furfey, 2014; Yancey and Forys, 2010), covered distance (Rolland et al., 2019), and kinematics of the flight close to the water surface (Blake, 1983, 1985; Withers and Timko, 1977). However, interactions with the coastal winds and wind gradients in their environment, and their effects on the black skimmer foraging behavior have received less consideration, although Zusi (1962) noted that while skimming, the black skimmers would change their wing beat frequency according to their flight angle with respect to the wind direction.

**Fig. 1.**
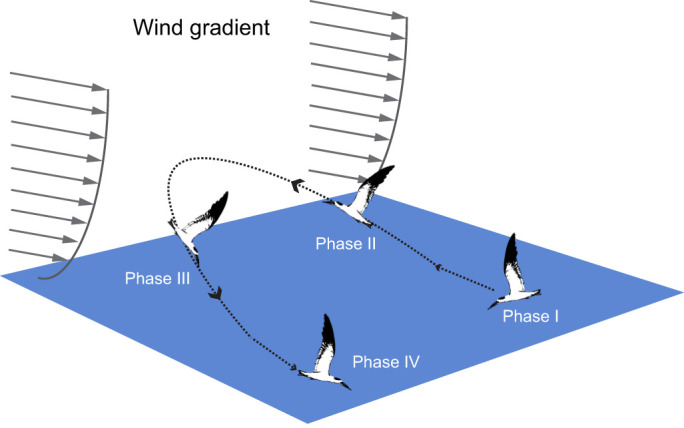
**Halfpipe skimming hypothesis.** The birds are skimming during phase I and phase IV regardless of wind direction. In phase II, the skimmers ascend facing the wind (upwind), decreasing their ground speed but increasing airspeed, and during phase III, the skimmers descend with the wind (downwind), increasing ground speed. Arrowheads show the flight direction. Phases I and IV occur in the skimming altitude condition identified in this paper, while phases II and III occur in the low and high altitude conditions.

We hypothesized that skimmers should take advantage of vertical wind speed gradients to further reduce their cost of foraging flight. We propose that, under what we hereafter will refer to as ‘halfpipe skimming’, skimmers that complete a feeding pass through a foraging patch (Fig. 1, phase I) should then fly upwind (in the air reference frame) while gaining altitude (Fig. 1, phase II), then turn downwind and descend gaining groundspeed (Fig. 1, phase III), and proceed to further feeding while flying level just above the water surface, using the enhanced groundspeed to produce lift (Fig. 1, phase IV). The hypothesized halfpipe skimming behavior, and other skimmer flight behaviors, are likely constrained by factors related to the feeding site and environment. For example, maximally exploiting the vertical wind gradient as an energy source requires the bird to ascend while flying perfectly upwind in the air reference frame and then descending while flying perfectly downwind. In real-world circumstances, doing so might lead the bird away from the feeding location, reducing foraging success, or require sharper turns that increase flight costs. However, as explored by Kempton et al. (2022), even suboptimal flight patterns through the wind gradient can provide an energy return for birds, and because some subsidy is presumably better than no subsidy, we expect that the skimmers will follow the hypothesized halfpipe skimming pattern. Furthermore, even if the energy gain is small, other options such as reversing the hypothesized pattern actually increase flight costs, so some alternative flight patterns might be actively detrimental.

In order to assess whether skimmers follow our halfpipe skimming predictions, we recorded black skimmers foraging in their natural habitat on the North Carolina coast, along with measurements of wind direction and wind speed to measure the flight direction, flight speed and kinetic energy of these birds during natural foraging flights.

## MATERIALS AND METHODS

### Field sites

Recordings of black skimmers (*Rynchops niger* Linnaeus 1758) were taken at two field sites on the North Carolina coast. A set of recordings was taken at the North Pond in the Pea Island National Wildlife Refuge in Rodanthe (35.72453 latitude, −75.49741 longitude) on 20 September 2020 between 17:30 h and 18:00 h (one recording session). A second set of recordings was taken at Shell Island in Wrightsville Beach (34.24331, −77.77175) on three different days (total of four recording sessions): 21 and 26 October 2021 between 17:00 h and 19:00 h, and on 8 April 2022 between 17:00 h and 18:00 h. Temperature, humidity and atmospheric pressure were collected at each recording session (Table 1). To the best of our knowledge the black skimmer colonies in North Pond and Shell Island were independent of each other. North Pond is a managed manmade impoundment open water area, with exposed mud flats and emerging vegetation. The recordings were taken from the Highway NC 12 side overlooking the pond close to the mud flat where the colony of about 50 birds was resting (Fig. S1A). Here, the colony was composed of breeding adults. Shell Island is a white sand beach, and the recordings were taken at an inlet on the beach leading to water canals and nesting habitat (Audubon North Carolina Coastal Sanctuary). In Shell Island, during the October recordings, the colony of about 200 individuals was composed of adults and juveniles. The black skimmers were found in this area right before migration. During the April recordings, the colony was composed of about 100 resident and migrating adults. In Shell Island, the recordings were taken looking at the sandy shore where the colonies were resting (Fig. S1B).

**Table 1.**
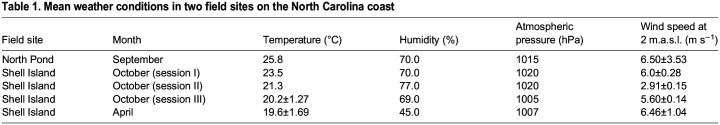
Mean weather conditions in two field sites on the North Carolina coast

### Recording and camera calibration

Three digital Canon EOS 6D digital SLR cameras equipped with 50 mm lenses were placed so that they had overlapping fields of view of the coastline and the skimmer colony. Videos were recorded at 29.97 frames s^−1^. The viewing direction of the lenses was measured with a compass and the distance between cameras was recorded. They were synchronized by visual events identified separately in each camera. They were then calibrated for 3D position reconstruction following the protocol from Corcoran and Hedrick (2019) via bundle adjustment using previously determined pinhole model lens parameters, and shared 2D information visible in at least two of the three cameras such as points along the coastline, boats, houses along the far coast and a subset of the flying skimmers. Scene scale was established from the distances between the three cameras, and calibration parameters were converted to direct linear translation (DLT) form for ease of analysis. The calibration was aligned to place the water surface at *Z*=0 with positive *Z* pointing upward and the *X* and *Y* directions forming the horizontal plane, with axes aligned to wind direction as described below. The maximum inter-camera distance was approximately 16 m and the birds tracked in this study were 6–145 m away from the cameras. Video coordinates were acquired with DLTdv (Hedrick, 2008) and bundle adjustment calibrations were performed using the MATLAB computer vision toolbox. Among all recordings, the mean DLT residual for the tracked birds was 0.832 pixels and the mean 95% confidence interval radius was 8.6 cm.

### Wind direction and wind gradient

For the recordings at Shell Island, a 36 cm windvane was placed in the sand at a height of 28 cm from the ground, located close to the coastline, and within the field of view of at least two of the cameras. The center post and tail of the windvane were tracked using DLTdv for the entirety of each video recording and used to determine the wind direction. This time-varying direction was then averaged for each separate recording. Manual wind direction was also taken with a compass at the beginning and end of each recording session to later corroborate wind direction with the digitized windvane points. For North Pond, we gathered the wind direction from meteorological data for 20 September 2020 and used the camera positions to find the wind vector.

**Fig. 2.**
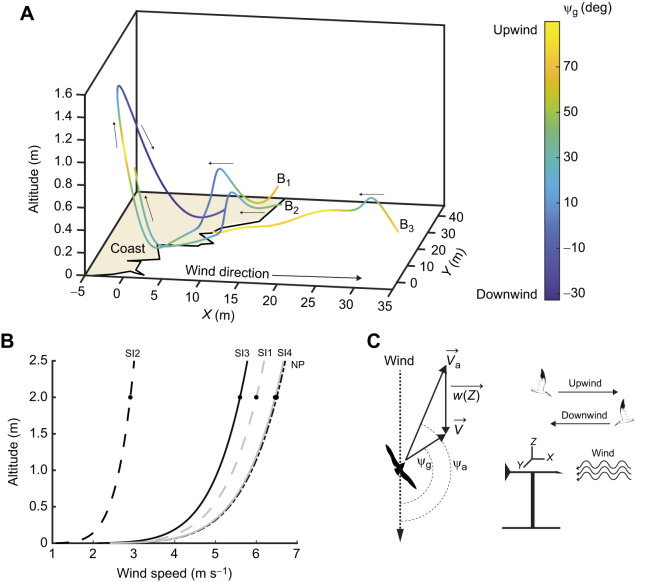
**Measurements and flight angle definition.** (A) Three bird tracks (B_1_, B_2_ and B_3_) with flight angle with respect to the wind in the ground reference frame (ѱ_g_) overlaid on tracks. Small arrows show flight direction. (B) Wind gradient reproduced from a 2 m wind speed measurement using Eqn 1 for North Pond (NP), and four recording sessions at Shell Island (SI). (C) Definition of upwind and downwind flight direction with respect to the wind vane, and representation of flight angle with respect to the wind in the ground reference frame (ѱ_g_) and air reference frame (ѱ_a_). 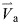, airspeed vector; 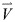, groundspeed vector; 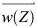, wind vector.

Wind speed was measured with a handheld anemometer (Model HHF142, Omega Engineering, Stamford, CT, USA) at the edge of the water on the coastline. For each recording session, five to six wind speed measurements were taken in a period of 30–60 s at each of the heights listed below; these values were then averaged to get a wind speed mean and standard deviation per height. In North Pond, measurements were taken at approximately 2 m from the ground, and in Shell Island they were taken at 0.035 m (radius of anemometer, the closest it could be placed to the ground), 1.0, 2.0 and 2.5 m for three of the recordings and at 0.035, 0.5, 1.0, 1.5, 2.0 and 2.7 m for one recording. To ensure a comparable gradient analysis among all sites, we used the following power law relationship to compute a wind gradient:
(1)

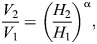
where *V*_2_ is the unknown wind speed at height *H*_2_, *V*_1_ is the measured wind speed at height *H*_1_ and α is the wind shear exponent. For our results, *H*_1_ was 2 m, *V*_1_ was measured as described above, and for α we used the canonical value of 0.143 (Bratton and Womeldorf, 2011; Schlichting and Gersten, 2000) (Fig. 2B). Additionally, for the Shell Island recordings, we computed an alternative gradient by using Eqn 1 to calculate a *V*_2_ value for each separate *V*_1_ measurement taken, then used the median of these *V*_2_ values as the basis for the analysis. Results from analyzing the Shell Island data only with this alternative gradient were similar to those from the analysis covering all sites and are presented in Tables S1–S3.

### Bird tracking

Black skimmers were manually digitized using DLTdv when skimming behavior was observed, and each bird was tracked for the entirety of their flight that was visible by at least two cameras (Fig. 2A; Fig. S2). Each bird track was then smoothed using a 4-pole digital Butterworth low-pass filter with a cutoff frequency of 0.5 Hz prior to further analysis. Instantaneous derivatives of position with respect to time were calculated by fitting a quintic spline to the smoothed data and differentiating the spline polynomial.

### Reference frames

To better understand how the birds used the environment, we considered their movements in the ground reference frame and the air reference frame using the wind gradient estimates. The wind direction (see ‘Wind direction and wind gradient’, above) was used to align the ground reference frame, such that movements in the increasing horizontal *X*-axis were downwind (in the direction of the wind) and those in the decreasing horizontal *X*-axis were upwind (against the wind) (Fig. 2C). Then, the flight angle with respect to the wind in the ground reference frame (ψ_g_) was computed directly from the ground reference frame *XYZ* velocity, 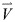 (Fig. 2C). ψ_g_ can be considered as the direction the birds are moving towards, and is directly observed by the cameras. Flight angle with respect to the wind in the air reference frame ψ_a_ was defined as the angle between the bird's airspeed vector (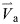) and the wind vector [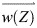] (Fig. 2C). ψ_a_ is the direction the bird flies with respect to the wind to achieve the course revealed as its trajectory in the ground reference frame. The air reference frame combines information from the cameras and the wind gradient.

Flight angles with respect to the wind (ψ_g_ and ψ_a_) were rotated so that perpendicular (crosswind) flights happen at 0 deg, completely upwind flights at 90 deg and completely downwind flights at −90 deg. This was done to facilitate interpretation of statistical analyses (see ‘Analysis of bird flight angle with respect to the wind’ and ‘Flight speed and kinetic energy analysis’, below).

### Flight speed and kinetic energy calculation

We calculated the groundspeed (*V*_g_) and airspeed (*V*_a_) for each tracked bird as:
(2)

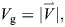




(3)

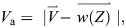



where 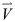 is the observed bird velocity in the ground reference frame and 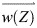 is the wind vector at different heights from the ground reconstructed from the gradient model and wind measurements at 2 m altitude.

From these different flight speeds, the mass-specific kinetic energy in the ground reference frame (*K*_g_) and air reference frame (i.e. with respect to the air, *K*_a_) were calculated as:
(4)

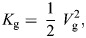

(5)

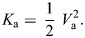


### Analysis of flight track data

Although our camera-based bird measurements provide precise tracking of position and movement in the ground reference frame, our knowledge of the exact wind environment experienced by an individual bird is much less certain and our wind measurements show variation around the recorded means, producing unavoidable variation in wind-associated analyses. For example, even if the skimmers fly perfectly upwind with respect to the instantaneous wind direction when ascending, our calculations based on average wind direction will show variation in the skimmer wind angle. For this reason, we avoided analyses of individual flight trajectories and instead used statistical analyses encompassing the entire dataset or large portions of it and mixed-effects statistical models that accommodate our data structure, including repeated samples at multiple levels: track, session and site.

### Analysis of bird flight angle with respect to the wind

We expected the altitude over the water surface to affect flight behavior and so we divided the data into three conditions: (1) flights in skimming position with a cutoff at *Z*≤0.20 m; (2) low flights at 0.20<*Z*≤0.40 m; and (3) high flights at 0.40<*Z*≤0.75 m. The cutoffs were selected based on percentage of observations, with 0.4 m being the third quantile and 0.75 m encompassing 90% of the data, and finally 0.2 m being the maximum altitude where black skimmers were observed feeding; it also was the median altitude in our recordings. We used this classification and the flight angle with respect to the wind from each track to find how the birds position themselves as they approach, fly over and leave the water surface. To assess the hypothesized relationships between flight angle with respect to the wind, ascending or descending behavior, and altitude under these three conditions, we used three linear mixed-effects models (one for each altitude condition) with 10,850, 5463 and 3831 observations (i.e. video frames), respectively. Each model included two fixed effects: vertical speed (*Z*′: ascending/descending flight) and altitude (*Z*), and nested random intercept and slope for site (North Pond and Shell Island), session and bird track. These random effects account for the inherent pseudoreplication of making multiple position measurements as part of each bird track and recording multiple bird tracks from each recording site and during each recording session. Each of the three models included data from both sites, all five recording sessions, and from 69, 70 and 64 tracks, respectively.

### Wind effectiveness number

Following Kempton et al. (2022), we also calculate a wind effectiveness number ε for the skimmer flights. For our dataset this was calculated as:
(6)

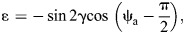
where γ is the bird's flight trajectory angle above (or below) horizontal. Because of the different conventions for ψ_a_ direction, Eqn 6 differs slightly from the equivalent in Kempton et al. (2022). We also used the total wind effectiveness number rather than the horizontal wind effectiveness number, as our camera data provide precise data on bird flight trajectories, and the near-shore environment with <10 cm waves (see below) makes vertical wind movement unlikely. As Kempton et al. (2022) show, ε>0 corresponds to energy harvesting and ε<0 corresponds to energy loss, both at a rate proportional to the magnitude of ε for equivalent wind gradients. For halfpipe skimming, we expect ε to have an average near 0 for flights at skimming altitude, becoming >0 at higher altitudes. We also expect an average >0 regardless of altitude when the skimmers are making rapid changes in altitude, defined as vertical velocity magnitude greater than the 80th percentile. Wind effectiveness results were evaluated using a mixed-effects model for ε with an intercept and random intercepts for site (North Pond and Shell Island), session and track.

### Flight speed and kinetic energy analysis

We compared *V*_g_ and *V*_a_ at the three flight conditions for upwind flights (flights angles between 0 and 90s) and downwind flights (flights angles between −90 and 0 deg) by relating the two in a mixed-effects model framework with nested random intercept and slope for site (North Pond and Shell Island), session and track to mitigate pseudoreplication within the dataset.

To explore the relationships between flight speed (*V*_g_ and *V*_a_) and flight angle with respect to the wind (ψ_g_ and ψ_a_), and kinetic energy (*K*_g_ and *K*_a_) and flight angle with respect to the wind, we used a set of linear mixed-effects models for each of the three altitude conditions described earlier. Each model included one fixed effect: flight angle (−90 to 90 deg with respect to the wind) and random effects as described above. For all kinetic energy analyses, we used flight data lying within the 95th percentile, ignoring extremely high energy results as likely errors.

### Energy extraction analysis

We evaluated the possibility of an energetic benefit from wind interactions through two methods. The first approach estimated the maximum energy available in theory to the skimmer based on the wind gradient, and the second focused on the groundspeed measurements at different altitude, as these are closest to our foundational data of skimmer flight positions through time. For the first estimate, we computed the mass-specific kinetic energy of a skimmer flying upwind in the skimming position with zero wind speed and airspeed equal to the skimmer minimum power speed. This result was then compared with the mass-specific air reference frame kinetic energy of a skimmer flying upwind with an airspeed of minimum power plus the wind speed estimate at 0.75 m over the ground, the 90th percentile of the recorded elevations. The difference between these values represents the energy gap between the speed needed to maintain flight and the air reference frame kinetic energy available if a skimming bird was instantly moved to an altitude typical of higher altitude flight in our recordings. As such, it represents a maximal estimate of the energy available but is not practically achievable, e.g. as a result of constraints on actual flight direction and costs related to climbing and turning flight.

Our second approach looked at the difference in average mass-specific kinetic energy in the ground reference frame between skimmers flying downwind in the high altitude condition and skimmers flying downwind in the skimming altitude condition, with the difference representing the wind gradient energy available to the birds. Because it is based on the overall average ground speed, this comparison assumes that the skimmer has come into equilibrium with the wind at both altitudes, i.e. that the skimmer's airspeed is similar in the two conditions. Full extraction of the gradient measured this way assumes that the skimmers come into equilibrium with the wind passively (by drag) rather than actively (by flapping).

## RESULTS

### Flight tracks, bird behavior and weather conditions

**Fig. 3.**
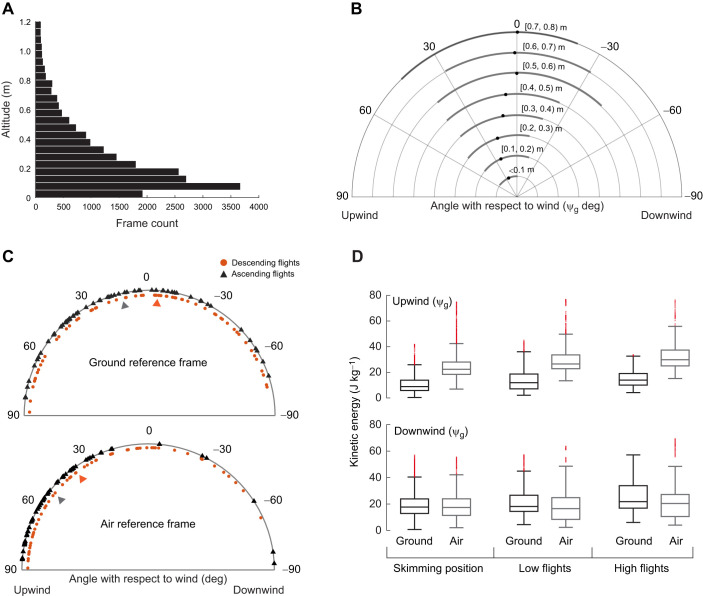
**Flight characteristics.** (A) Altitude distribution of the black skimmer positions by frame. (B) Median ψ_g_ for all tracks at increasing altitude intervals with the interquartile ranges. (C) Median ψ_g_ (top) and ψ_a_ (bottom) for each bird track for ascending (*Z*′>0.01 m s^−1^) and descending (*Z′*<−0.01 m s^−1^) flights, at high flight conditions (0.4<*Z*≤0.75 m). Arrowheads indicate the median flight direction for all tracks. (D) *K*_g_ and *K*_a_ for each flight condition (*Z*≤0.2 m, 0.2<*Z*≤0.4 m and 0.4<*Z*≤0.75 m) for upwind and downwind flights in the ground reference frame (ψ_g_) (linear mixed-effect models looking at the relationship between kinetic energy and flight angle with respect to the wind can be found in Table S4). Box plots show median, upper and lower quartiles and interquartile range. Outliers ranged from 0.21% to 6.80% of total data, and omission of these outliers did not change the results.

A total of 70 flights (North Pond: 15, Shell Island: 55) were tracked. In these tracks, the skimmers spent most of their foraging time below 0.4 m of altitude (75% of the data) with 25% of the data being at or below 0.1 m over the water surface, which we found was the mean altitude when skimming was observed in the videos (Fig. 3A). Observation of skimming was not possible at longer distances because of video resolution limits, so more distant birds flying within 0.1 m of the water may have also been skimming. Track durations averaged 10.7 s, with a standard deviation of 6.0 s. The flight path lengths were 57.4±36.0 m (mean±s.d.) in the ground reference frame and 74.2±45.5 m in the air reference frame.

Weather conditions between days and sites varied slightly, with conditions in North Pond being warmer and windier (Table 1). Wind speeds ranged from 1.2 to 9.33 m s^−1^ over the entirety of the dataset. As noted earlier, in the main text, we report results based on a simple wind gradient model using the wind speed measurement at a height of 2 m, thus allowing direct comparison of data from the two field sites. Water surface conditions near the coastline at both sites remained calm throughout all recording sessions, with low waves (≤10 cm). Complementary results from the multiple-sample gradient are presented in Tables S1–S3.

### Descriptive quantification of flight behavior

Across the 70 flight tracks recorded in this study, the average ground speed (*V*_g_) was 5.33±1.55 m s^−1^ (*n*=70). Using the same mean of means quantification scheme, average air speed (*V*_a_) was 6.70±1.90 m s^−1^, average ψ_g_ was 18.89±31.52 deg (i.e. slightly upwind) while average ψ_a_ was 42.71±24.65 deg. Average movement speed in the horizontal *X* direction was −0.75±2.56 m s^−1^ (*n*=70), denoting a slight upwind tendency, while average movement speed in the crosswind direction was −0.22±2.90 m s^−1^ and movement in the vertical direction averaged −0.01±0.08 m s^−1^, with an 80th percentile magnitude of 0.37 m s^−1^ and a 95th percentile magnitude of 0.81 m s^−1^.

### Flight angle with respect to the wind

For flights at skimming altitude (*Z*≤0.2 m) and low flights (0.20<*Z*≤0.40 m), we found that the flight altitude and flight angle with respect to the wind were significantly correlated in the ground reference frame (linear mixed-effects models: *P*<0.01 and *P*<0.05, respectively; Table 2). The coefficients in these relationships show that birds flying at a higher altitude over the water surface tended to fly in a more downwind direction than birds closer to the water surface (Fig. 3B). In contrast, in the air reference frame, flight altitude and angle with respect to the wind were uncorrelated at these two lower altitude conditions.

**Table 2.**
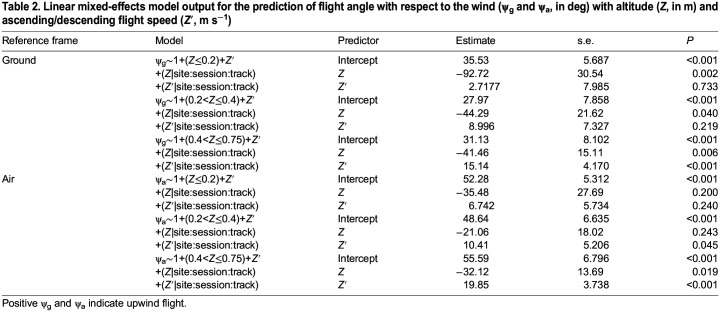
**Linear mixed-effects model output for the prediction of flight angle with respect to the wind (ψ_g_ and ψ_a_, in deg) with altitude (*Z*, in m) and ascending/descending flight speed (*Z*′, m** **s^−1^)**

For the high altitude flight condition (0.40<*Z*≤0.75 m), the altitude relationship described above was still significantly present in the ground reference frame (*P*<0.01; Table 2) and also present in the air reference frame (*P*<0.05). However, it was overshadowed by a highly significant correlation between angle with respect to the wind and descending/ascending flight speed (*Z*′) in both reference frames (linear mixed-effects models: *P*<0.001; Table 2). In this case, the coefficients show that birds descending towards the water surface tended to fly more downwind and those ascending tended to fly more upwind (Table 2, Fig. 3C). This correlation was also weakly significant in the air reference frame in the low altitude flight condition data (linear mixed-effects models: *P*<0.05; Table 2). Coefficients relating flight angle with respect to the wind to ascending and descending flights were also larger in the air reference frame (Table 2).

### Wind effectiveness

For all skimmer flight data at skimming altitude, the average wind effectiveness ε was 0.00 and not significantly different from 0. This was also the case for all flight data in the low and high altitude conditions.

For ascending and descending flight where the magnitude of the vertical velocity vector (*Z′*) was above its 80th percentile, 0.37 m s^−1^, we found that the average ε was 0.06, significantly different from zero (*P*=0.0012). Interestingly, ascending and descending flights had quite distinct values for ε. For skimmers with an ascent speed >0.37 m s^−1^, ε was 0.14, significantly different from 0 (*P*<0.001), but for skimmers with a descent speed <−0.37 m s^−1^, ε was −0.02 (*P*=0.001), denoting slight energy loss to the wind gradient during descents. Ascent and descent flight speed profiles were similar; the 80th percentile of descent speed was −0.38 m s^−1^, while the 80th percentile of ascent speed was 0.37 m s^−1^.

### Airspeed and kinetic energy versus groundspeed and kinetic energy

To illustrate how flight speed changes in relation to the reference frame and demonstrate that wind speed magnitudes were sufficient to produce substantial differences between airspeed and groundspeed, we compared groundspeed (*V*_g_) with airspeed (*V*_a_) over the three altitude conditions. As expected mathematically, birds flying at upwind angles (0–90 deg) had a greater *V*_a_ than *V*_g_ at all three altitude conditions (skimming position, low flights and high flights) (*P*<0.001 for all conditions; Table 3). This pattern changed when birds were flying at downwind angles (−90 to 0 deg), with *V*_g_ becoming greater than *V*_a_ (*P*<0.02 in the high and low flight conditions; Table 3). These speed differences are recapitulated in mass-specific kinetic energy differences among the air and ground reference frames (Fig. 3D).

**Table 3.**
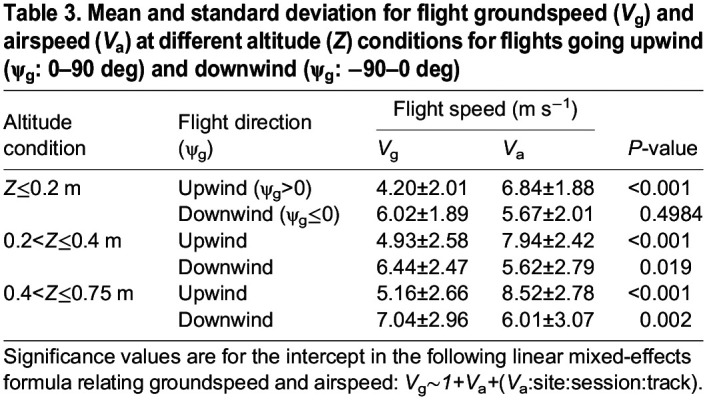
**Mean and standard deviation for flight groundspeed (*V*_g_) and airspeed (*V*_a_) at different altitude (*Z*) conditions for flights going upwind (ψ_g_: 0–90** **deg) and downwind (ψ_g_: −90–0** **deg)**

To further isolate the effects of the flight angle with respect to the wind (ψ_g_ and ψ_a_) on the flight speed and kinetic energy, we used the linear mixed-effects models (Table 4; Table S4). For *V*_g_, we found that *ψ_g_* and *ψ_a_* were correlated with flight speed at the three altitude conditions (skimming position, low flights, high flights) (Linear mixed-effects models: *P*<0.001; Table 4). As expected, the coefficients show *V*_g_ decreasing as the birds approach a completely upwind flight angle of 90 deg with respect to the wind (Table 4). This leads to the same results for *K_g_*, where we would expect to see a decrease in kinetic energy as the birds approach a complete upwind flight at all three altitude conditions (Linear mixed-effects models: *P*<0.001; Table S4; Fig. 3D). In contrast, we found that airspeed was mostly uncorrelated with ψ_g_ or ψ_a_ at all altitude conditions (linear mixed-effects models: *P*>0.05, Table 4; Tables S2 and S3). However, we found significant correlations of *K*_a_ with ψ_g_ and ψ_a_ during the high altitude flight conditions above 0.4 meters from the water such that kinetic energy tended to increase for upwind angles (linear mixed-effects model: *P*<0.05; Table S4; Fig. 3D).

**Table 4.**
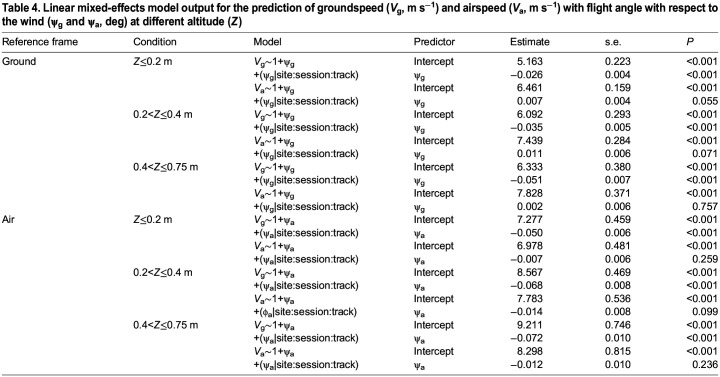
**Linear mixed-effects model output for the prediction of groundspeed (*V*_g_, m** **s^−1^) and airspeed (*V*_a_, m** **s^−1^) with flight angle with respect to the wind (ψ_g_ and ψ_a_, deg) at different altitude (*Z*)**

### Energy extraction

As described in the Materials and Methods, we estimated the energy available for extraction as the difference in air reference frame kinetic energy for a skimmer flying at minimum power at the skimming position, and estimated gradient wind speed with a skimmer flying at minimum power speed plus the median wind speed in the high altitude condition. For this calculation, we took the minimum power speed to be 5.8 m s^−1^, which was the average downwind airspeed in our results averaged across the three altitude conditions (Table 3). Downwind speeds were selected because cost of transport considerations and the need to make progress through the water for skimming may cause skimmers flying upwind to increase airspeed. The estimated wind speed at 0.75 m altitude averaged across the five recording sessions was 4.78 m s^−1^, whereas it was 3.58 m s^−1^ at the skimming altitude of 0.1 m s^−1^ and thus the theoretical mass-specific energy available to skimmers from the wind gradient and the altitude range used in practice was 7.7 J kg^−1^.

Our second estimate of energy extraction used the observed mean ground reference frame kinetic energy at the high altitude condition and skimming condition, moving downwind in both cases. Using data from Table 3, this second calculation estimated the energy available as 6.7 J kg^−1^. Because this analysis uses groundspeed at the observed flight angles, rather than estimating ground speed at a perfectly downwind angle, it already accounts for the actual flight angles.

## DISCUSSION

Here, we investigated how birds may use their aerial environment to subsidize their flight costs. Specifically, we proposed that black skimmers use the wind gradient to their advantage during foraging flight, using it to gain energy and speed while changing flight direction by ‘halfpipe skimming’, which we defined as: flying upwind in the air reference frame and gaining altitude to take advantage of the increasing wind speed in the vertical wind gradient, followed by turning in a downwind direction (again in the air reference frame) and descending, gaining ground speed from the tailwind and gravitational potential energy. After descending, the birds then feed at low altitude under no major influence from the wind direction as they fly close to the water where wind speeds are lower and flight costs are reduced by the ground effect. Our results support parts of this hypothesis: skimmers gaining altitude do so in an upwind direction relative to skimmers losing altitude, but the difference in flight angle during ascent and descent indicated inefficient exploitation of the wind gradient.

The flight behavior we recorded could be the result of other factors that were not considered in this study, such as topography and fish aggregation. Water currents can affect the fish behavior (Becker et al., 2016; Haak et al., 2019) and combined with the coastline, these factors determine the location of the food patch, constraining the flight direction of the foraging skimmers. Thus, the precise foraging strategy employed by skimmers will result from a multi-component trade-off between aerial environment, aquatic environment and prey in these environments.

### Halfpipe skimming

As expected for halfpipe skimming, in the air reference frame, flight direction with respect to the wind was correlated with change in altitude such that birds gaining altitude flew more into the wind, while descending birds flew more downwind (Table 2). These correlations were significant for flights above 0.2 m of altitude and higher. Because skimmers at low altitude are potentially feeding while trying to remain within the ground effect (Blake, 1985; Rayner, 1991; Su et al., 2013) and setting their flight direction based on feeding patch orientation, we were not surprised to see them only respond to wind direction while ascending and descending and not when in the skimming position.

The wind effectiveness number (ε) analysis sheds further light on how the skimmers interact with the wind gradient. We initially hypothesized that ε would be positive for skimmers flying in the low and high altitude conditions. However, because calculation of ε includes the upward or downward trajectory angle of the bird, and skimmer flight is, on average, nearly horizontal in all altitudes, ε was instead nearly 0 at all altitudes. Furthermore, energy gain from the wind gradient cannot occur without the bird changing altitude, so a focus on ε when the birds were ascending or descending proved more informative. Consistent with the results from Table 2, ascending birds had a positive ε of about 0.14, showing that they tended to fly upwind while gaining altitude. However, descending birds, rather than turning fully downwind, only turned downwind relative to the ascent, and tended to fly in a nearly cross-wind direction with an average ε of about −0.02. Combined, these ascending and descending results for ε are consistent with positive energy extraction that is limited by other factors previously mentioned. We believe this is the most likely reason that skimmers did not further optimize ε and energy extraction from the wind gradient; doing so would require a more downwind flight direction with an associated fast groundspeed that would move them away from the feeding location.

We estimated a potential energetic benefit for halfpipe skimming of 7.7 J kg^−1^ using a wind gradient-centered approach, and 6.6 J kg^−1^ using the observed ground speed data. If conducted at the end of a 5 s long skimming bout, this amounts to a benefit of approximately 1.3–1.5 W kg^−1^. Blake (1985) modeled the aerodynamic power curve for skimmers feeding in ground effect and found a minimum flight cost of 56 W kg^−1^ for a 357 g skimmer. Thus, the halfpipe subsidy would reduce flight costs by a further 2.5% below this minimum. Another way of considering the magnitude of the halfpipe skimming benefit is by comparison to the potential energy differences between the altitude conditions studied here. The difference in altitude between skimming position and the upper margin of the high altitude flight conditions is 0.65 m, or 6.4 J kg^−1^ of potential energy. Thus, the halfpipe skimming subsidy theoretically available from the observed behavior is almost exactly enough to let the skimmers move from skimming to the high altitude condition at no additional cost.

Even though our results are generally consistent with the halfpipe skimming strategy and suggest an energetic benefit, they cannot demonstrate how much of this benefit the birds receive as the skimmers are also flapping – a necessity, given that the expected energetic subsidies are much less than the cost of flight computed by Blake (1985). Furthermore, the skimmers could extract more energy from the available gradient. For example, moving from skimming to a higher altitude than the observed 0.75 m would make a larger difference in wind speeds and thus more energy available, and turning more sharply into the wind when ascending would access that energy more quickly. Thus, the halfpipe skimming behavior may represent a compromise between several considerations including gathering an environmental flight subsidy but also effectively exploiting the available foraging patch.

### Other black skimmer–wind interactions

In general, we found that black skimmers as a group remained in the same location in the ground reference frame, with a mean ground reference frame flight direction close to zero (Fig. 3B,C). Skimmers also maintained broadly similar airspeeds regardless of flight direction with respect to the wind (Table 4) and thus we found that (as mathematically required) black skimmers flying upwind had a greater airspeed compared with their groundspeed, with the opposite occurring during downwind flights. This demonstrates that the wind speeds in these recordings were sufficient to affect flight behavior. Furthermore, as a necessary corollary to these results, skimmers spent most of their time flying upwind in the air reference frame. We also found that within each altitude condition there was a trend for skimmers further from the water surface to fly in a more downwind direction, even while the overall time average was for upwind flight. This trend was significant in all three altitude conditions for the ground reference frame, but only in the high altitude condition (0.4 to 0.75 m) in the air reference frame. Thus, skimmers will generally use the high altitude condition when flying downwind, and fly closer to the water surface where wind speeds are lower when they go upwind.

### Flight at skimming altitude

Most of the flight time for the birds in this study occurred below 0.2 m altitude, and the birds were skimming or preparing to skim at 0.1 m. Flight in this altitude condition was also mostly upwind, so the skimmers were flying upwind for most of their foraging time. Even at this low altitude, groundspeed was significantly affected by the wind direction, and upwind flights reduced groundspeed (and ground kinetic energy). This may further improve the energetics of skimming by reducing the hydrodynamic drag on the submerged mandible, which already has riblets that serve this function (Martin and Bhushan, 2016), while ground effect decreases the aerodynamic power required, as previously described for skimmers (Blake, 1983, 1985; Withers and Timko, 1977) and in open-water feeding bats (Johansson et al., 2018). Finally, upwind flights are needed to repeatedly exploit the same food patch and by staying close to the water surface the birds can move at a lower flight cost as they are flying within the low wind speed portion of the wind gradient, so the effects of losing ground kinetic energy are minimized, as has been analyzed by Alerstam et al. (2019) in foraging flights for other birds.

### Conclusions

Foraging black skimmers tune their flight direction with respect to the wind when ascending to exploit a flight subsidy from the vertical wind gradient and avoid working against the aerial environment. However, the subsidy likely amounts to less than 2.5% of their flight costs and efficiently exploiting it appears to be a secondary consideration compared with others such as using flight directions that keep the bird within the food patch.

## Supplementary Material

10.1242/jexbio.246855_sup1 Supplementary information
