# Supplementary material for: Wind gradient exploitation during foraging flights by black skimmers (Rynchops niger)
Source: J Exp Biol. 2024 Aug 22;227(16):jeb246855. doi: 10.1242/jeb.246855 (PMC11418178; doi:10.1242/jeb.246855)
Supplement: Supplementary information [file jexbio-227-246855-s1.pdf]

A

B

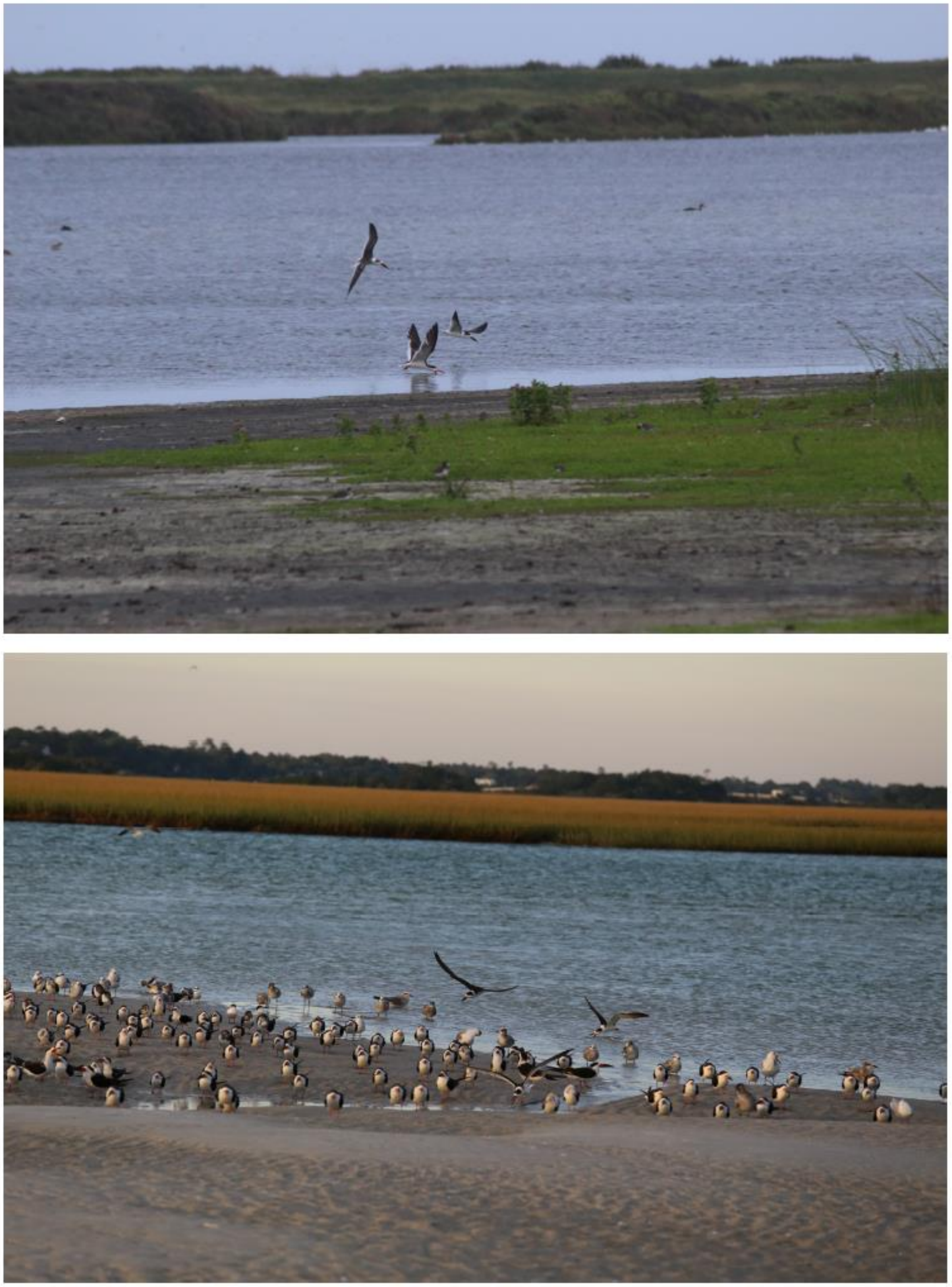

Fig. S1. Both field sites. (A) North Pond, picture with permission of Sonja Friman. (B) Shell Island, picture with permission of Jonathan Rader.

Journal of Experimental Biology • Supplementary information

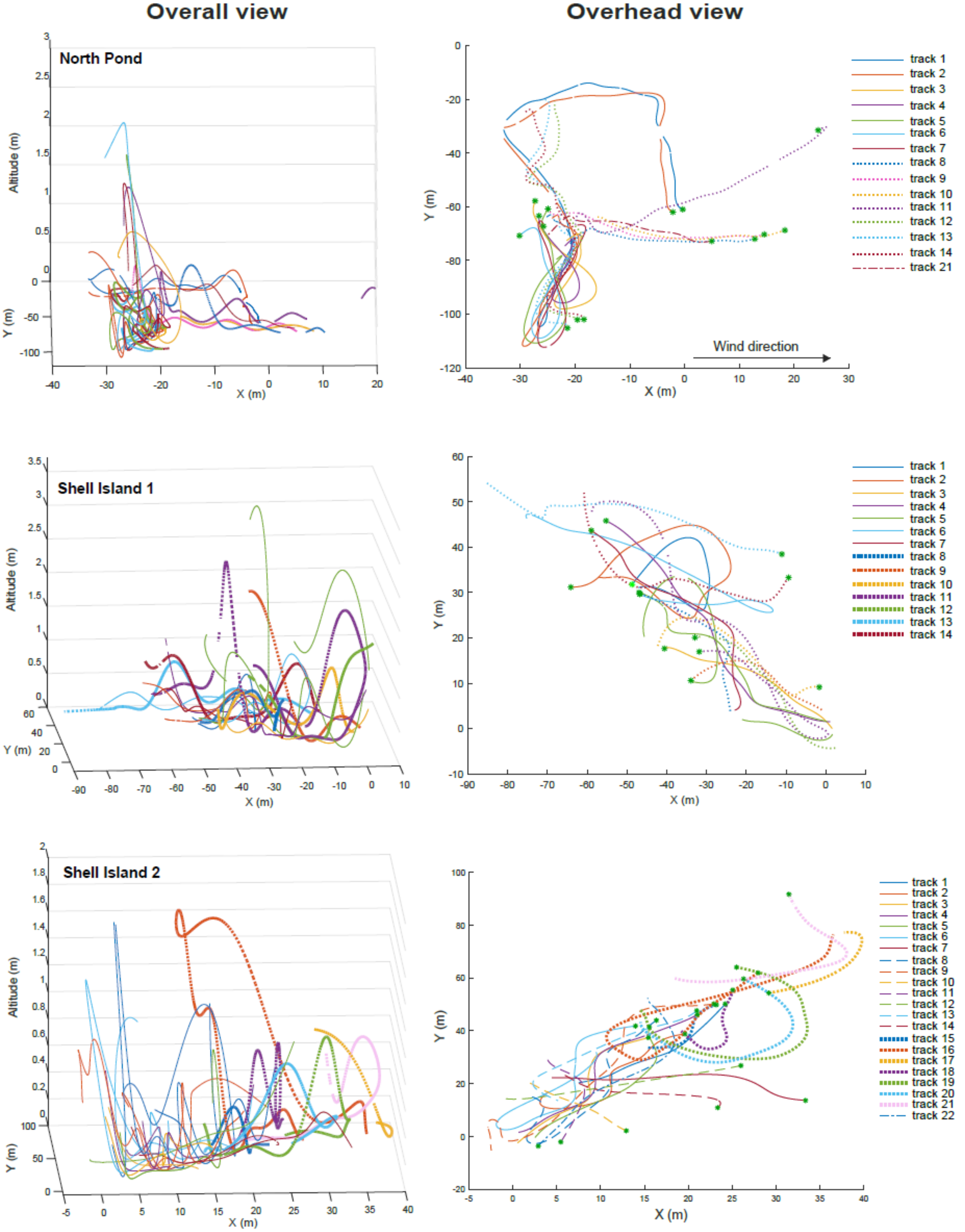

Journal of Experimental Biology • Supplementary information

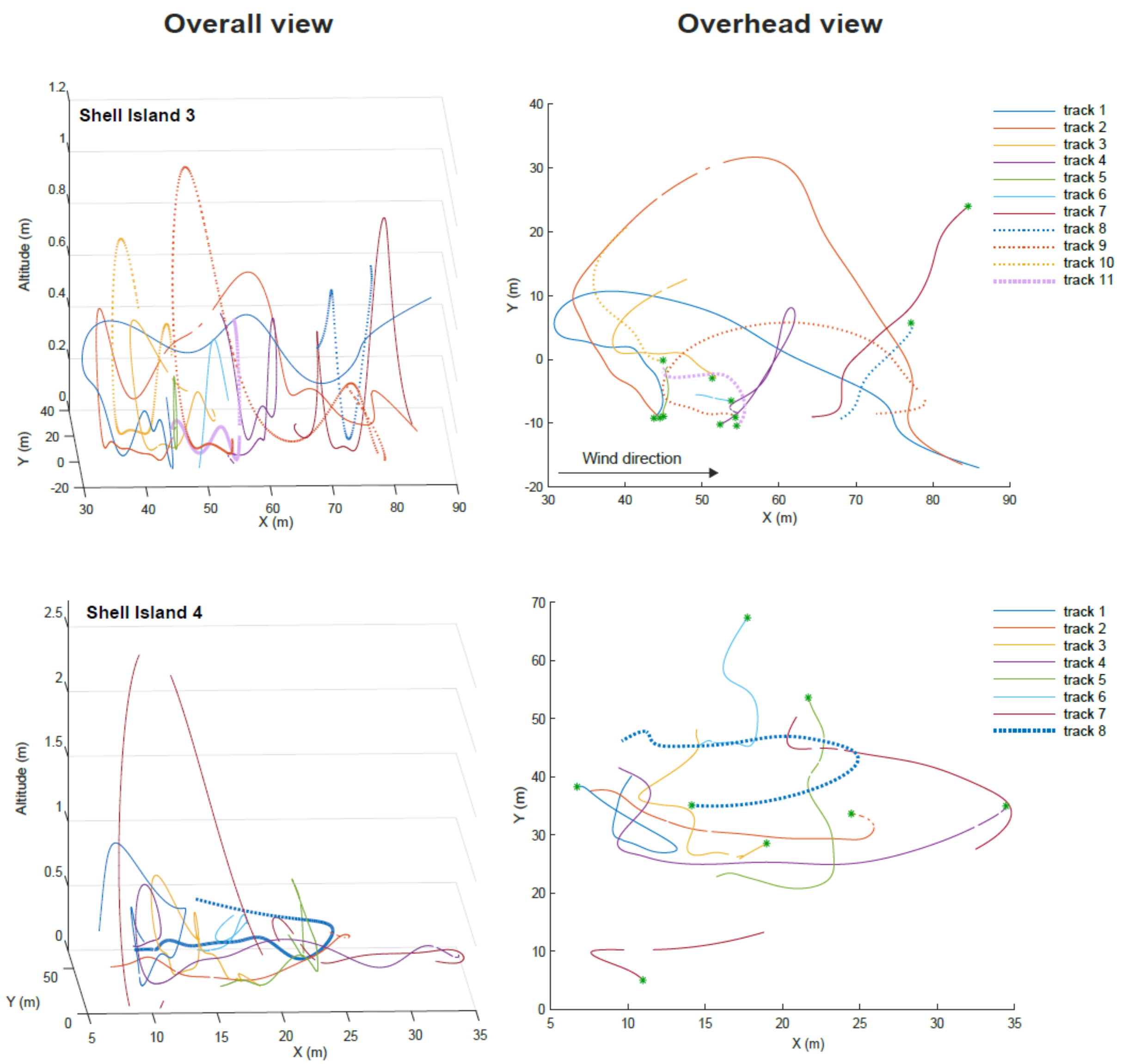

Fig. S2. All skimmer tracks. Overhead and overall view of all tracks by session. Green asterisks on the overhead view represent the start of each track.

Journal of Experimental Biology • Supplementary information

Table S1. Linear mixed-effects model output for the prediction of flight angle with respect to the wind in the multi-sample gradient air reference frame (ψ_{as}) in degrees with altitude in meters (Z) and ascending/descending flight speed in meters per second (Z'). ψ_{as} was defined as the angle between the bird's airspeed vector ($\overrightarrow{V_a}$) and the wind vector ($\overrightarrow{w(Z)_s}$) based on the measured wind gradient calculated from observations at multiple heights. Complement to Table 2 in the main text.

Model	Predictor	Estimate	s.e.	*P*
ψ_{as} ~1+(Z≤0.2)+Z' +(Z|site:session:track) +(Z'|site:session:track)	Intercept	52.60	6.654	<0.001
	Z	-51.49	35.50	0.147
	Z'	3.907	6.927	0.572
ψ_{as} ~1+(0.2<Z≤0.4)+Z' +(Z|site:session:track) +(Z'|site:session:track)	Intercept	42.89	7.897	<0.001
	Z	-7.474	21.28	0.725
	Z'	8.357	6.115	0.172
ψ_{as} ~1+(0.4<Z≤0.75)+Z' +(Z|site:session:track) +(Z'|site:session:track)	Intercept	60.67	7.281	<0.001
	Z	-47.60	14.43	<0.001
	Z'	19.58	4.640	<0.001

Table S2. Linear mixed-effects model output for the prediction of groundspeed (V_g) and airspeed based on the measured wind gradient calculated from observations at multiple heights ($V_{as} = \left|\vec{V} - \overrightarrow{w(Z)_s}\right|$, where $\overrightarrow{w(Z)_s}$ is the wind vector as a function of altitude given by the same gradient) in meters per second with flight angle with respect to the wind in the air reference frame (ψ_{as}) in degrees at different altitude (Z) conditions. Complement to Table 4 from the main text.

Condition	Model	Predictor	Estimate	s.e.	*P*
Z ≤ 0.2 m	V_g ~1+ ψ_{as} +(ψ_{as}|site:session:track)	Intercept	7.704	0.607	<0.001
		ψ_{as}	-0.056	0.007	<0.001
	V_{as} ~1+ ψ_{as} +(ψ_{as}|site:session:track)	Intercept	7.350	0.664	<0.001
		ψ_{as}	-0.015	0.008	0.055
0.2 < Z ≤ 0.4 m	V_g ~1+ ψ_{as} +(ψ_{as}|site:session:track)	Intercept	7.610	0.350	<0.001
		ψ_{as}	-0.051	0.006	<0.001
	V_{as} ~1+ ψ_{as} +(ψ_{as}|site:session:track)	Intercept	6.669	0.427	<0.001
		ψ_{as}	0.002	0.007	0.708
0.4 < Z ≤ 0.75 m	V_g ~1+ ψ_{as} +(ψ_{as}|site:session:track)	Intercept	7.621	0.318	<0.001
		ψ_{as}	-0.050	0.005	<0.001
	V_{as} ~1+ ψ_{as} +(ψ_{as}|site:session:track)	Intercept	6.428	0.423	<0.001
		ψ_{as}	0.009	0.007	0.175

Journal of Experimental Biology • Supplementary information

Table S3. Linear mixed-effects model output for the prediction of airspeed (V_{as}) in meters per second with flight angle with respect to the wind (ψ_g) in degrees at different altitude (Z) conditions. Complement to Table 4 from main text and Table S2.

Condition	Model	Predictor	Estimate	s.e.	*P*
$Z \leq 0.2$ m	V_{as} ~1+ ψ_g	Intercept	6.466	0.218	<0.001
	+(ψ_g|site:session:track)	ψ_g	0.003	0.005	0.491
$0.2 < Z \leq 0.4$ m	V_{as} ~1+ ψ_g	Intercept	7.074	0.190	<0.001
	+(ψ_g|site:session:track)	ψ_g	0.010	0.005	0.079
$0.4 < Z \leq 0.75$ m	V_{as} ~1+ ψ_g	Intercept	7.098	0.228	<0.001
	+(ψ_g|site:session:track)	ψ_g	0.014	0.006	0.018

Journal of Experimental Biology • Supplementary information

Table S4. Linear mixed-effects model output for the prediction of the mass-specific kinetic energy in the ground reference frame (K_g) and air reference frames (K_a and K_{as}) in Joules per kilogram with flight angle with respect to the wind (ψ_g, ψ_a, and ψ_{as}) in degrees at different altitude (Z) conditions. $K_{as} = ½V_{as}^2$ and is the mass-specific kinetic energy in the air reference frame based on the measured wind gradient calculated from observations at multiple heights.

	Condition	Model	Predictor	Estimate	s.e.	*P*
Ground Reference Frame Angles (θ)	Z ≤ 0.2 m	K_g ~1+ ψ_g	Intercept	15.414	1.169	<0.001
		+(ψ_g|site:session:track)	ψ_g	-0.166	0.028	<0.001
		K_a ~1+ ψ_g	Intercept	22.36	1.006	<0.001
		+(ψ_g|site:session:track)	ψ_g	0.017	0.029	0.555
		K_{as} ~1+ψ_g	Intercept	22.95	1.327	<0.001
		+(ψ_g|site:session:track)	ψ_g	-0.017	0.039	0.667
	0.2 < Z ≤ 0.4 m	K_g ~1+ ψ_g	Intercept	18.86	1.140	<0.001
		+(ψ_g|site:session:track)	ψ_g	-0.189	0.026	<0.001
		K_a ~1+ψ_g	Intercept	26.59	0. 997	<0.001
		+(ψ_g|site:session:track)	ψ_g	0.048	0.030	0.107
		K_{as} ~1+ ψ_g	Intercept	25.77	1.011	<0.001
		+(ψ_g|site:session:track)	ψ_g	0.035	0.034	0.312
	0.4 < Z ≤ 0.75 m	K_g ~1+ ψ_g	Intercept	19.44	1.338	<0.001
		+(ψ_g|site:session:track)	ψ_g	-0.209	0.032	<0.001
		K_a ~1+ ψ_g	Intercept	28.15	1.223	<0.001
		+(ψ_g|site:session:track)	ψ_g	0.080	0.033	0.017
		K_{as} ~1+ ψ_g	Intercept	26.75	1.135	<0.001
		+(ψ_g|site:session:track)	ψ_g	0.082	0.040	0.040
Air Reference Frame Angles (ψ_a and ψ_{as})	Z ≤ 0.2 m	K_g ~1+ ψ_a	Intercept	22.36	1.482	<0.001
		+(ψ_a|site:session:track)	ψ_a	-0.218	0.027	<0.001
		K_g ~1+ ψ_{as}	Intercept	23.32	1.760	<0.001
		+(ψ_{as}|site:session:track)	ψ_{as}	-0.234	0.033	<0.001
		K_a ~1+ ψ_a	Intercept	22.22	2.138	<0.001
		+($\psi_a$site:session:track)	ψ_a	-0.006	0.038	0.866
		K_{as} ~1+ ψ_{as}	Intercept	24.59	3.226	<0.001
		+(ψ_{as}|site:session:track)	ψ_{as}	-0.059	0.052	0.255
	0.2 < Z ≤ 0.4 m	K_g ~1+ ψ_a	Intercept	27.64	1.542	<0.001
		+($\psi_a$site:session:track)	ψ_a	-0.259	0.026	<0.001
		K_g ~1+ ψ_{as}	Intercept	25.60	1.474	<0.001
		+(ψ_{as}|site:session:track)	ψ_{as}	-0.234	0.028	<0.001
		K_a ~1+ ψ_a	Intercept	24.66	2.111	<0.001
		+($\psi_a$site:session:track)	ψ_a	0.020	0.037	0.586
		K_{as} ~1+ ψ_{as}	Intercept	21.65	2.124	<0.001
		+(ψ_{as}|site:session:track)	ψ_{as}	0.039	0.044	0.375

Journal of Experimental Biology • Supplementary information

$0.4 < Z \leq 0.75$ m	$K_g \sim 1+\psi_a + (\psi_a\|site:session:track)$	Intercept	28.90	1.915	<0.001
		ψ_a	-0.240	0.031	<0.001
	$K_g \sim 1+\psi_{as} + (\psi_{as}\|site:session:track)$	Intercept	28.07	1.791	<0.001
		ψ_{as}	-0.231	0.033	<0.001
	$K_a \sim 1+\psi_a + (\psi_a\|site:session:track)$	Intercept	22.05	3.063	<0.001
		ψ_a	0.108	0.053	0.041
	$K_{as} \sim 1+\psi_{as} + (\psi_{as}\|site:session:track)$	Intercept	19.39	2.907	<0.001
		ψ_{as}	0.118	0.058	0.044

Journal of Experimental Biology • Supplementary information
